# Prognostic role of early D-dimer level in patients with acute ischemic stroke

**DOI:** 10.1371/journal.pone.0211458

**Published:** 2019-02-01

**Authors:** Jing Zhang, Lin Liu, Jie Tao, Yanlin Song, Yimeng Fan, Maling Gou, Jianguo Xu

**Affiliations:** 1 Department of Neurosurgery and National Clinical Research Center for Geriatrics, West China Hospital, Sichuan University, Chengdu, Sichuan, PR China; 2 Department of Neurosurgery, Neijiang Hospital of Traditional Chinese Medicine, Neijiang, Sichuan, PR China; 3 Department of Biotherapy, Cancer Center, West China Hospital, Sichuan University and Collaborative Innovation Center for Biotherapy, Chengdu, Sichuan, PR China; Charite Universitatsmedizin Berlin, GERMANY

## Abstract

**Object:**

The purpose of our study was to assess the prognostic role of early D-dimer level in patients with acute ischemic stroke (AIS).

**Methods:**

The included patients’ D-dimer levels have to be tested within 24 hours from stroke onset. Poor functional outcome was defined as modified Rankin Scale (mRS) ≥3. The endpoints included recurrence on 5-day diffusion-weighted imaging, 30-day mRS ≥3, 30-day mortality and 90-day mRS ≥3. Regarding to each endpoint, odds ratios (ORs) and 95% confidence intervals (CIs) were calculated to assess the prognostic role of D-dimer in patients with AIS.

**Results:**

A total of 2,479 patients were included. The results showed that elevated D-dimer levels were associated with recurrence on 5-day diffusion-weighted imaging (OR = 2.28, 95% CI = 1.32–3.95), 30-day mRS≥3 (OR = 1.59, 95% CI = 1.37–1.85), 30-day mortality (OR = 1.92, 95% CI = 1.27–2.90) and 90-day mRS≥3 (OR = 1.61, 95% CI = 1.05–2.46).

**Conclusions:**

In conclusion, for patients with AIS, higher D-dimer level within 24 hours from stroke onset was associated with recurrence on 5-day diffusion-weighted imaging, mortality at 30 days, and poor functional outcome at both 30 days and 90 days. However, more studies are warranted to clarify this issue.

## Introduction

Stroke is a leading cause of disability and mortality around the world [1]. There are 2.5 million new stroke cases every year and 7.5 million stroke survivors in China [2]. Ischemic stroke, which represents around 80% of all strokes, is a result of blockade of the cerebral blood vessels [3]. The diagnosis of acute ischemic stroke (AIS) is usually based on history, physical examination and brain imaging (noncontrast CT or MRI) [4]. Thrombolysis in the therapeutic window (4.5 hours after stroke onset [5]) is beneficial for patients, however, few patients are eligible for thrombolytic therapy [6]. Many factors were found to be associated with the outcome of AIS, such as age, gender, stroke severity, atrial fibrillation, congestive heart failure, diabetes and so on [7, 8]. However, it is still difficult to predict outcome even for experienced neurologists [9]. It is useful to detect new predictors to help manage AIS [10].

Recently, many studies have investigated the association between levels of plasma hemostatic markers and AIS outcomes [10–13]. Among these markers, the role of D-dimer in AIS was a hot spot of research. D-dimer, a degradation product of cross-linked fibrin, is a biomarker of the fibrinolytic and coagulation system [14]. Since D-dimer is resistant to ex vivo activation, relatively stable, and has a long half-life, it has certain advantages over thrombin measures [15]. Some researchers reported that increased D-dimer level independently predicted poor outcome in patients with AIS [12, 16]. However, some researchers did not reach such conclusions [17, 18]. Therefore, the purpose of this study was to systematically evaluate the prognostic role of D-dimer level in patients with AIS through performing a meta-analysis.

## Methods

### Search strategy

We performed this study according to the guidelines for meta-analyses [19]. All relevant studies were identified by searching the PubMed, Embase, Web of Science and Cochrane Library prior to Oct 29th, 2017, and no start date was applied. The search terms included: (‘Stroke’ OR ‘Cerebral Infarction’ OR ‘Brain Infarction’ OR ‘Brain Ischemia’) AND ‘D-dimer’ AND (‘Prognosis’ OR ‘Outcome’ OR ‘Mortality’) (S1 Table). Additional studies were obtained by checking the references of relevant literature. Article languages included Chinese and English.

### Study selection

Two investigators (JZ and LL) independently performed the study selection, and disagreements were resolved by consensus. The inclusion criteria included: (1) the patients were diagnosed with acute ischemic stroke, admitted within 24h of onset and received standard therapy; (2) blood sample is collected within 24h of onset to test the D-dimer level; (3) patients were adequately followed up for survival outcomes, functional outcomes or other events; (4) enough data was provided to assess the prognostic role of D-dimer in patients with acute ischemic stroke. Those studies were excluded: reviews, case reports, conference abstracts, letters, unrelated articles, and studies without sufficient data. Studies obtaining blood samples after receiving thrombolysis were also excluded.

### Data extraction

Two investigators (JZ and JT) independently extracted relevant data from included studies, with any disagreements being discussed. The main data was odds ratio (OR) for poor outcomes with 95% confidence interval (CI). Adjusted ORs or ORs from multivariate analyses were extracted over unadjusted ones or ORs from univariate analyses. The study and patient characteristics included first author, patient source, publication year, number of patients, sex, mean age and other characteristics.

### Study quality assessment

The Newcastle–Ottawa Scale (NOS) quality assessment criteria were used to assess the quality of the studies [20]. The scale assessed three aspects of the studies: subject selection: 0 to 4; subject comparability: 0 to 2; and exposure: 0 to 3. The NOS scores ranged from 0 to 9. Studies scoring 7 or more were regarded as high-quality ones.

### Statistical analysis

Poor functional outcome was defined as modified Rankin Scale (mRS) ≥3. LogOR and variance, which were converted from OR and 95% CI, were pooled in the software. Forest plots were outlined to assess the pooled prognostic role of D-dimer in patients with AIS. The pooled ORs were considered significant if the p values were <0.05 or the 95% CIs did not overlap one. The between-study heterogeneities were also evaluated, with P<0.10 or I^2^>50% implying significant heterogeneity [21]. Due to the differences of characteristics across studies, random effect models were used in combining the data [22]. Sensitivity analyses were performed to assess the contribution of every study to heterogeneity by excluding studies one by one. Publication bias was evaluated by Begg’s test and p>0.05 was considered that there was no potential publication bias. All the statistical analyses were performed by STATA 11.0 (STATA Corporation, College Station, TX).

## Results

### Literature research

**Fig 1:**
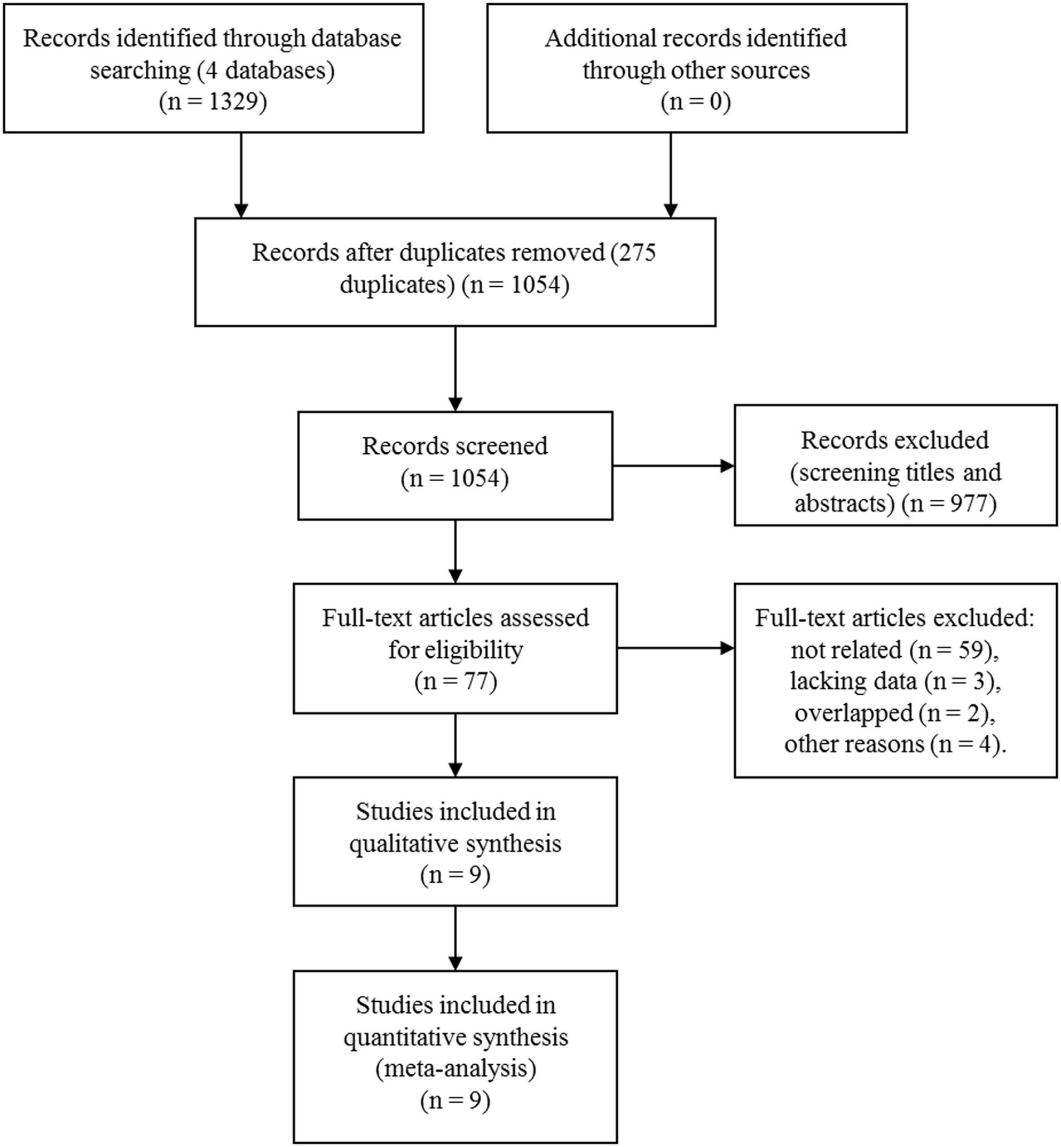
Selection process of studies.

The initial literature research yielded 1,329 studies from the four databases. No additional records were found from other sources. Among them, 275 duplicated articles were removed and 1,054 studies were further screened. After screening for the titles and abstracts, 977 studies were excluded according to the inclusion criteria and exclusion criteria. The rest 77 studies were assessed in full text and 64 were excluded due to unrelated (n = 59), lacking enough data (n = 3) or overlapped research (n = 2). One study examined the D-dimer level after receiving intravenous thrombolysis in AIS patients, so it was excluded [23]. In another study, 28.2% of the patients received thrombolytic therapy and blood samples were collected after thrombolysis, so it was also excluded [24]. Two more studies assessed the D-dimer level later than 24h from stroke onset and were also excluded [7, 25]. Eventually, 9 articles were included in our study [6, 8–13, 26, 27]. The study inclusion process was shown in Fig 1.

### Study characteristics

The baseline characteristics of the 9 included studies were shown in Table 1. They were published from 2009 to 2016. They were conducted in seven different countries. A total of 2,479 patients were included (mean 275, female 1,026, male 1,453). The mean ages of the patients in the studies were around 60–70 years old. All the patients were admitted within 24 hours from stroke onset, and the blood samples for D-dimer tests were collected within 24 hours from stroke onset. Six studies reported the stroke subtypes by Trial of Org 10172 in Acute Stroke Treatment (TOAST). The D-dimer assay included immunoturbidimetric assay (ITA) and enzyme-linked immunosorbent assay (ELISA). Most studies did not report the cut-off value of D-dimer. The endpoints included recurrence on 5-day diffusion-weighted imaging (5D DWI recurrence), poor functional outcome at 30 days (30D mRS ≥3), mortality at 30 days (30D mortality), and poor functional outcome at 90 days (90D mRS ≥3). All the ORs were adjusted or calculated from multivariate analyses. In the NOS quality assessment, five studies scored 9 and four studies scored 8, which demonstrated they were high-quality studies.

**Table 1:** Characteristics of the included studies.

Author	Year	Country	N (F/M)	Mean age (Yrs)	Onset to admission	Sample time	TOAST Subtypes (LAA/CE/SVO/other)	D-dimer assay	Cut-off	Endpoints	Univariate*	Multivariate*	Quality score
Kang	2009	South Korea	153 (54/99)	64.6	≤24 hours	≤24 hours	28.1%/21.6%/ 35.3%/15.0%	ITA	per 1 Log D-dimer	5D DWI Recurrence	positive	positive	9
Abdel Ghani	2011	Egypt	50 (17/33)	60.6	≤24 hours	≤24 hours	20.0%/42.0%/ 34.0%/4.0%	ITA	NR	5D DWI Recurrence	positive	positive	8
Welsh	2009	UK	180 (96/84)	69	≤24 hours	≤24 hours	NR	ELISA	NR	30D mRS≥3	positive	positive	9
Wang	2016	China	1173 (422/751)	66.7	≤24 hours	≤24 hours	23.1%/5.4%/ 42.9%/28.6%	ITA	NR	30D mRS≥3	positive	positive	9
Shibazaki	2009	Japan	335 (125/210)	72.3	≤24 hours	≤24 hours	8.1%/39.7%/ 14.3%/37.9%	NR	>1.4 mg/L	30D mortality	positive	NS	9
Uestuendag	2010	Turkey	91 (49/42)	64.5	≤24 hours	≤24 hours	25.3%/28.6%/ 18.7%/27.5%	ITA	NR	30D mortality	positive	negative	8
Sienkiewicz-Jarosz	2009	Poland	54 (27/27)	73.3	≤24 hours	≤24 hours	NR	ITA	NR	90D mRS≥3	positive	positive	8
Whiteley	2012	UK	268 (156/112)	74.4	≤24 hours	≤24 hours	NR	ELISA	75th to the 25th centile	90D mRS≥3	positive	NS	9
Park	2013	South Korea	175 (80/95)	66	≤24 hours	≤24 hours	29.7%/28.0%/ 21.7%/20.6%	ELISA	NR	90D mRS≥3	positive	NS	8

N (F/M): number of patients (Female/Male), Yrs: years, TOAST subtypes: proportion of main stroke subtypes by Trial of Org 10172 in Acute Stroke Treatment (TOAST) (large-artery atherosclerosis, LAA/cardioembolism, CE/small-vessel occlusion, SVO/stroke of other determined etiology or stroke of undetermined etiology), NR: not reported, ITA: immunoturbidimetric assay, ELISA: enzyme-linked immunosorbent assay, 5D DWI recurrence: recurrence on 5-day diffusion-weighted imaging, mRS: modified Rankin Scale, 30D mRS≥3: poor functional outcome at 30 days, 30D mortality: mortality at 30 days, 90D mRS≥3: poor functional outcome at 90 days

* positive: higher D-dimer level was associated with worse outcome; negative: higher D-dimer level was associated with better outcome; NS: not significant; Kang adjusted for baseline infarct volume, acute multiple infarcts on initial DWI, time from onset to initial MRI, stroke subtype, anticoagulation versus antiplatelets or none; Abdel Ghani adjusted for infarction volume, onset to initial DW-MRI, atherosclerosis, acute multiple infarction; Welsh adjusted for age, stroke subtype, admission Scandinavian Stroke Scale score, atrial fibrillation on ECG, C-Reactive protein, fibrinogen, IL-6, leukocyte count, vWF antigen, factor VIIIc, factor Ixc, prothrombin F1+2, thrombin-antithrombin complex; Wang adjusted for age, sex, hypertension, diabetes, smoking, hyperlipidemia; Shibazaki adjusted for atrial fibrillation, National Institutes of Health Stroke Scale score, glucose, albumin, brain natriuretic peptide; Uestuendag adjusted for atrial fibrillation, left ventricular hypertrophy, left atrial thrombus, stroke subtype, length of stay in acute hospital; Sienkiewicz-Jarosz adjusted for National Institutes of Health Stroke Scale score, Barthel Index, atrial fibrillation, neuron-specific enolase, S100B protein, C-Reactive protein, glucose, taurine; Whiteley adjusted for age, initial National Institutes of Health Stroke Scale score; Park adjusted for age, initial National Institutes of Health Stroke Scale score.

### Outcomes analyses

**Fig 2:**
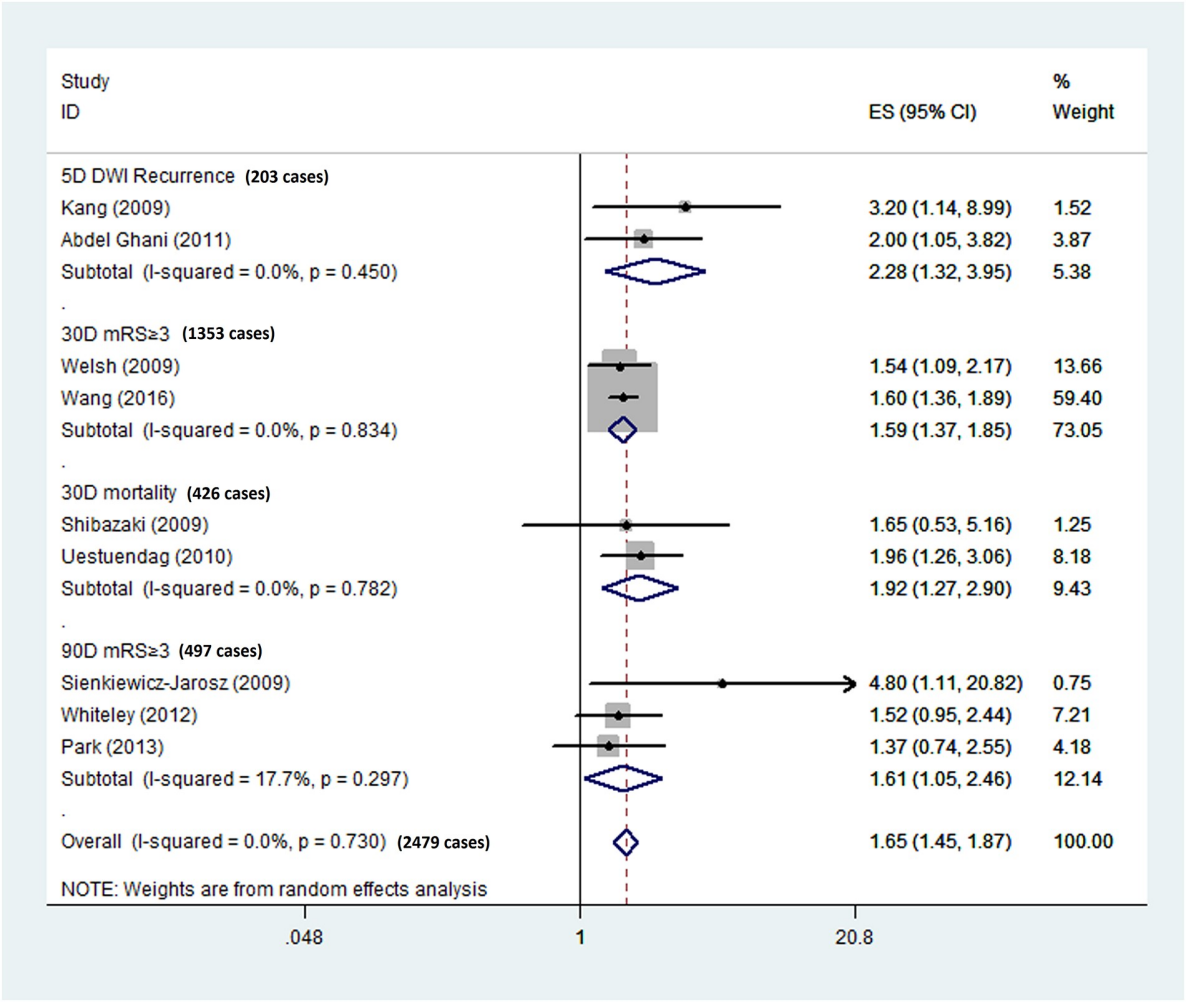
Forest plots. Pooled odds ratios (ORs) of higher D-dimer levels for different endpoints in patients with acute ischemic stroke.

Two studies used 5D DWI recurrence as the endpoint [13, 27]. The pooled OR of the two studies showed that elevated D-dimer levels were associated with recurrence on 5-day diffusion-weighted imaging (OR = 2.28, 95% CI = 1.32–3.95) in patients with AIS (Fig 2). No significant heterogeneity was found between the two studies (I^2^ = 0.0%, p = 0.450).

Two studies used 30D mRS ≥3 as the endpoint [10, 12]. The pooled OR of the two studies showed that elevated D-dimer levels were associated with poor functional outcome at 30 days (OR = 1.59, 95% CI = 1.37–1.85) in patients with AIS (Fig 2). No significant heterogeneity was found between the two studies (I^2^ = 0.0%, p = 0.834).

Two studies used 30D mortality as the endpoint [8, 9]. The pooled OR of the two studies showed that elevated D-dimer levels were associated with mortality at 30 days (OR = 1.92, 95% CI = 1.27–2.90) in patients with AIS (Fig 2). No significant heterogeneity was found between the two studies (I^2^ = 0.0%, p = 0.782).

Three studies used 90D mRS ≥3 as the endpoint [6, 11, 26]. The pooled OR of the three studies showed that elevated D-dimer levels were associated with poor functional outcome at 90 days (OR = 1.61, 95% CI = 1.05–2.46) in patients with AIS (Fig 2). Still, no significant heterogeneity was found between the studies (I^2^ = 17.7%, p = 0.297).

### Publication bias

No significant publication bias was found as to the 2 studies examining 5D DWI recurrence, 2 studies examining 30D mRS ≥3, 2 studies examining 30D mortality and the 3 studies examining 90D mRS ≥3.

## Discussion

The purpose of our study was to evaluate the prognostic role of early D-dimer level in patients with AIS. A meta-analysis was performed to summarize the evidence, and 9 studies were included. To our knowledge, this is the first meta-analysis in this field. Our results demonstrated that increased D-dimer level was associated with recurrence on 5-day diffusion-weighted imaging, mortality at 30 days, and poor functional outcome at both 30 days and 90 days.

D-dimer represents the activation of coagulation and fibrinolysis [12]. The D-dimer levels were found to be higher in many disorders in which the coagulation system is activated such as AIS [9, 13]. Increased D-dimer level is associated with higher risk of deep venous thrombosis, myocardial infarction, pulmonary thromboembolism, stroke and other diseases [10, 28]. In AIS patients with different subtypes, the levels of D-dimer are also different [7, 27]. Its level is the highest in cardio-embolic stroke and lowest in lacunar subtype [27]. The reason might be that, the thrombus in cardio-embolic stroke is mostly caused by blood stasis and is fibrin-rich [27], while the thrombi in lacunar stroke are too small to produce detectable elevated D-dimer levels [29].

Several mechanisms were raised to explain the correlation between elevated D-dimer level and worse outcome in AIS. Elevated D-dimer level may reflect the ongoing thrombus formation in brain vessels [30]. Thus, D-dimer may be a marker of systemic hypercoagulability [31]. Besides, D-dimer may activate the inflammatory process [32]. It was reported that D-dimer could activate monocyte to release proinflammatory cytokines such as interleukin-6 [33]. However, more studies are needed to further address the underlining mechanism.

In this study, we only included studies that collected blood samples within 24h of stroke onset, since the D-dimer level may dynamically change at different time points after AIS [26]. Sienkiewicz-Jarosz et al. [26] assessed the levels of D-dimer at the time of admission, and at 24 and 72 hours after stroke onset. They found that the D-dimer levels in the patients with poor outcome (mRS ≥3) were significantly higher than the levels in patients with good outcome at the three time points. For the patients with poor outcome, the D-dimer levels increased moderately at 24 hours and increased dramatically at 48 hours. For the patients with good outcome, the D-dimer levels also increased at 24 hours, but decreased at 48 hours. They also found that increased D-dimer levels on admission and at 24 hours were significantly associated with poor outcome; however, the association was not significant as to the D-dimer level at 48 hours. So, more studies exploring the association between D-dimer lever on admission, which is not influenced by therapy, and the outcome in AIS are needed to confirm our findings. Also, it would be interesting to explore the role of serial D-dimer levels on AIS prognosis.

Since thrombolytic treatment have a great impact on outcome [5], it would be of great interest to evaluate the prognostic role of D-dimer level in patients receiving thrombolytic treatment and patients not receiving thrombolytic treatment, respectively. After systematic literature research, only one study examined the prognostic role of D-dimer level in patients receiving thrombolytic treatment [23]. Hsu et al. [23] included patients admitted within 4.5 hours from stroke onset and the patients all received intravenous thrombolysis. They found that a higher level of D-dimer was significantly associated with an unfavorable outcome and the occurrence of symptomatic ICH. However, the D-dimer level was assessed within 24 hours from stroke onset. The D-dimer level could have been influenced by thrombolysis. Some researchers investigated patients not receiving thrombolytic treatment, and found D-dimer to be a promising prognostic biomarker [10, 12]. However, Park et al. [6] did not reach such a conclusion. Thus, more studies are needed to further address this issue.

There are some limitations in our study. Firstly, this meta-analysis included only a small number of studies. The number of studies in each endpoint was also limited. Therefore, the results need to be interpreted with caution and more studies are needed. Secondly, the characteristics of the patients in the studies were different, for example, the patients received different therapies. Thirdly, the majority of the patients were of Asian origin, but it is unable to perform subgroup analysis (Asians vs. non-Asians) due to the limited study number in each endpoint. Thus, the generalizability of the results still needs more research. Furthermore, although between-study heterogeneities and publication bias were not present among each endpoint, they are major concerns for meta-analysis and should not be excluded completely. Besides, the cut-off values in six studies were not reported, and the cut-off values in the other three studies were different. Although the three studies adopted different endpoints and were not in the same combined group, it is still a major limitation of this study. So our study just demonstrated a trend that higher D-dimer level was associated with worse outcomes. Only if the data were analyzed in a commonly used model, we will be able to get more meaningful results from the pooled analyses. In the future, much more studies are needed to find out the optimal cut-off value of D-dimer to guide its clinical use in prognosis.

In conclusion, our results showed that, for patients with AIS, higher D-dimer level was associated with recurrence on 5-day diffusion-weighted imaging, mortality at 30 days, and poorer functional outcome at both 30 days and 90 days. This readily accessible and cheap marker may help guide the management of AIS. However, due to the limited study number and heterogeneity among patients’ characteristics, more studies are warranted to further clarify this issue.

## Supporting information

S1 Table Detailed search criteria for Pubmed. (DOCX)

S2 Table PRISMA 2009 checklist. (DOC)
