# Supplementary material for: Prognostic role of early D-dimer level in patients with acute ischemic stroke
Source: PLoS One. 2019 Feb 1;14(2):e0211458. doi: 10.1371/journal.pone.0211458 (PMC6358072; doi:10.1371/journal.pone.0211458)
Supplement: S1 Table — (DOCX) [file pone.0211458.s001.docx]

Supporting Table 1: detailed search criteria for Pubmed

| Database | Search strategies |
| --- | --- |
| PubMed | 1. “Stroke” [Mesh] OR “Brain Ischemia” [Mesh] OR “Brain Infarction” [Mesh] OR “Cerebral Infarction” [Mesh] OR Cerebral Ischemia  2. “fibrin fragment D” [Mesh] OR fibrin fragment D1 dimer OR fibrin fragment DD OR D-dimer  3. “prognosis” [Mesh] OR outcome OR survival OR mortality  4. 1 AND 2 AND 3 |
